# Geschichte der chirurgischen Behandlung von Herzrhythmusstörungen in Deutschland: Chirurgische Therapie ventrikulärer Tachykardien sowie supraventrikulärer Tachykardien, insbesondere Präexzitationssyndromen (WPW)

**DOI:** 10.1007/s00399-024-01012-2

**Published:** 2024-02-28

**Authors:** Helmut U. Klein, Hans-Joachim Trappe, Günter Frank

**Affiliations:** 1 Hannover, Deutschland; 2 Dülmen, Deutschland; 3 Lübeck-Travemünde, Deutschland

**Keywords:** Kammertachykardie, Prä- und intraoperatives Mapping, Endokardinzision, Endokardresektion, Wolff-Parkinson-White-Syndrom, Ventricular tachycardia, Pre- and intraoperative mapping, Endocardial incision, Endocardial resection, Wolff-Parkinson-White syndrome

## Abstract

Die Geschichte der Behandlung ventrikulärer Tachykardien durch chirurgische Verfahren ist kurz; sie dauerte nicht länger als 15 Jahre von etwa 1978 bis 1993. Grundsätzlich sind zwei chirurgische Verfahren zu unterscheiden. Die *indirekten* Verfahren mit Resektion einer Infarktnarbe ohne elektrophysiologische Untersuchung und die *direkten* Verfahren mit gezielter Endokardinzision („encircling endocardial ventriculotomy“). In Deutschland haben Ostermeyer, Breithardt und Seipel (Düsseldorf) 1979 erstmals über intraoperatives, elektrophysiologisches Mapping bei ventrikulären Tachykardien (VT) berichtet; 1981 hat dann die Gruppe in Hannover (Frank, Klein) über erste Ergebnisse bei der chirurgischen Therapie von Kammertachykardien berichtet. 1984 konnten Ostermeyer, Breithardt und Borggrefe zeigen, dass eine nur partielle Endokardinzision deutlich günstiger und weniger Ventrikel schädigend (8 % vs. 46 %) ist als eine komplette zirkuläre Inzision. 1987 berichtete die Düsseldorfer Gruppe über Behandlungsergebnisse von 93 Patienten. Nach 5 Jahren waren 77 % der Patienten ohne VT-Rezidiv, die Gesamtmortalität nach einem Jahr war 11 %, nach 5 Jahren 30 %. Die Hannover Gruppe berichtete 1992 über 147 Patienten, die mit endokardialem Resektionsverfahren behandelt wurden; die Gesamtmortalität nach 3 Jahren war 27 %, VT-Rezidive traten bei 18 % der Überlebenden auf.

Die Geschichte der chirurgischen Verfahren bei supraventrikulären Tachykardien (SVT), insbesondere dem Wolff-Parkinson-White(WPW)-Syndrom ist noch kürzer als die der Chirurgie für VT. Bereits 1969 berichteten Sealy, Gallagher und Cox über die ersten Fälle einer chirurgischen Intervention beim WPW-Syndrom über einen endokardialen Zugang bei kardioplegischem Herzstillstand. 1984 berichteten Guiraudon und Klein über ein neues Verfahren mit epikardialem Zugang zum akzessorischen Bündel ohne Kardioplegie bei lateral lokalisierten Leitungsbahnen. Auch in Deutschland führten seit 1980 die Gruppen in Düsseldorf (Ostermeyer, Seipel, Breithardt, Borggrefe) und seit 1981 die Hannover Gruppe (Frank, Klein und Kallfelz) operative Verfahren bei WPW-Syndrom durch. Borggrefe berichtete 1987 über 18 operierte Patienten mit WPW-Syndrom und Vorhofflimmern. Nach 2 Jahren blieben 14 von 18 Patienten ohne Tachykardie Rezidive; 1989 berichteten Frank, Klein und Kallfelz (Hannover) über 10 operierte Kinder (2−14 Jahre) unter Anwendung der Kryoablationstechnik. Zwischen 1984 und 1992 wurden in Hannover insgesamt 120 Patienten mit SVT, meist WPW-Syndrom, operiert; nach 42 Monaten hatten 12 Patienten ein SVT-Rezidiv. Bei der Reoperation verstarben 2 Patienten.

## Abstract (en)

The history of surgical treatment of ventricular tachycardias (VT) is short, lasting from 1978 until 1993. “Indirect procedures” with infarct scar resection were performed without electrophysiologic studies, whereas “direct procedures” consisted of either complete endocardial incisions (“encircling endocardial ventriculotomy”) or large endocardial resections (“endocardial peel-off” technique) after precise epicardial and endocardial mapping procedures. In Germany, the first to report on intra-operative electrophysiologic mapping for VT treatment were Ostermeyer, Breithardt and Seipel in 1979. In 1981, the Hannover group (Frank, Klein) published their first results of surgical treatment of VT. In 1984, Ostermeyer et al. demonstrated that a partial endocardial incision resulted in more beneficial results with less myocardial damage (8% versus 46%) than applying a complete encircling incision. In 1987, the Düsseldorf group reported treatment results of 93 patients. After 5 years, 77% had no VT recurrence, while total mortality after 1 year was 11% and after 5 years 30%. In 1992, the Hannover group reported results of 147 patients after endocardial resection for VT. Total mortality after 3 years was 27%; recurrence of VT events occurred in 18% of the surviving cohort.

The history of surgical procedures for supraventricular tachycardia (SVT), in particular Wolff-Parkinson-White (WPW) syndrome, is even shorter than that of surgery for VT. As early as 1969, Sealy, Gallagher and Cox reported the first cases of surgical intervention for WPW syndrome via endocardial access in cardioplegic arrest. In 1984, Guiraudon and Klein reported on a new procedure with epicardial access to the accessory bundle without cardioplegia in laterally localised conduction pathways. In Germany, too, the groups in Düsseldorf (Ostermeyer, Seipel, Breithardt, Borggrefe) from 1980 and the Hannover group (Frank, Klein and Kallfelz) from 1981 performed surgical procedures for WPW syndrome. In 1987, Borggrefe reported on 18 patients with WPW syndrome and atrial fibrillation who had undergone surgery. After 2 years, 14 of 18 patients had no recurrences of tachycardia; in 1989, Frank, Klein and Kallfelz (Hannover) reported on 10 children (2−14 years) operated on using the cryoablation technique. Between 1984 and 1992, a total of 120 patients with SVT, mostly WPW syndrome, were operated on in Hannover; after 42 months, 12 patients had a recurrence of SVT. Two patients died during the reoperation.

## *Teil 1*: Chirurgische Therapie ventrikulärer Tachykardien

Der Zeitraum, in dem in Deutschland Herzrhythmusstörungen mit herzchirurgischen Verfahren therapiert wurden – oder anders ausgedrückt – als Elektrophysiologen und Herzchirurgen gemeinsam tachykarde Herzrhythmusstörungen behandelten, erstreckt sich etwa über 12 Jahre, von 1980 bis 1992. Warum wurde die Herzchirurgie involviert, welche Verfahren wurden angewandt, welche Ergebnisse wurden erzielt, und warum wurden diese chirurgischen Methoden wieder aufgegeben? Diesen Teil der Geschichte der Rhythmologie wollen wir in Erinnerung bringen.

Tachykarde Herzrhythmusstörungen, sowohl atriale als auch ventrikuläre Tachykardien, insbesondere die lebensbedrohlichen Formen und die Probleme des plötzlichen, arrhythmiebedingten Herztodes konnten bis in die 1970er Jahre nur durch Antiarrhythmika behandelt werden, obwohl die Ergebnisse nicht zufriedenstellend waren. Es gab Procainamid, Ajmalin und Verapamil zur akuten Terminierung von Tachykardien, zur Langzeitbehandlung blieb nur das nicht unproblematische Amiodaron. Alle anderen Antiarrhythmika, die in Studien getestet wurden, erwiesen sich als unbrauchbar. Bis etwa 1990 wurde der implantierbare Kardioverter-Defibrillator (ICD) nur bei relativ wenigen Patienten zur Sekundärprävention des plötzlichen Herztodes (PHT) angewendet.

Die Möglichkeit der Behandlung einer ventrikulären Tachykardie durch Resektion eines ventrikulären Aneurysmas wurde bereits 1959 von Couch [1] berichtet. Aber erst nach der Einführung der kardialen Katheterisierung, der Entwicklung von elektrophysiologischen Untersuchungen und dem intraoperativen Mapping erschien dann 1975 eine Arbeit von G. Fontaine über drei chirurgische Behandlungen mittels rechtsventrikulärer Ventrikulotomie nach epikardialem Mapping bei Patienten mit einer rechtsventrikulären Kardiomyopathie [2]. Damit begann ein neues Kapitel der Arrhythmiebehandlung. Vorausgegangen waren bereits tierexperimentelle Studien zur epikardialen Erregungsausbreitung in der Amsterdamer Gruppe um Dirk Durrer, Hein Wellens und Michiel Janse, sowie Albert L. Waldo in New York und später Birmingham, AL, USA. In wenigen Jahren bildeten sich einige Zentren, in denen Verfahren zum prä- und intraoperativen Mapping und damit auch chirurgische Therapien von Tachykardien entwickelt wurden.

So signalisierte W. Sealy von der Duke-Universität in Durham, NC, der bereits durch seine chirurgische Behandlung des Wolff-Parkinson-White(WPW)-Syndroms bekannt war, 1979, dass die direkte chirurgische Behandlung von Arrhythmien das „letzte für den Chirurgen zu betretende Gebiet der Kardiologie sei“ [3]. Wie viele derartiger Voraussagen der „Weisen“ hatte er Recht, was die folgenden 10 Jahre betraf. Dann widmete sich die Herzchirurgie anderen Gebieten, und die Arrhythmiebehandlung entwickelte sich weiter in den Händen der Kardiologen, d. h. der sich selbst ausbildenden Elektrophysiologen mit neuen Mappingverfahren und der Entwicklung der Katheterablationen.

In der zweiten Hälfte der 1970er Jahre entwickelte sich die programmierte Ventrikelstimulation, nicht nur zum Testen der Auslösbarkeit einer Tachykardie, sondern auch zur Vorhersage der Wirksamkeit verordneter Antiarrhythmika. Allerdings war schnell klar, dass eine Nichtauslösbarkeit kein sicherer Beweis für eine verlässliche Suppression von Tachykardierezidiven war.

Nach den ersten Berichten zur chirurgischen Behandlung von Tachykardien bei arrhythmogener rechtsventrikulärer Kardiomyopathie der französischen Gruppe um G. Fontaine und G. Guiraudon [4] erschienen dann sehr bald auch Arbeiten zur chirurgischen Behandlung von ventrikulären Tachykardien bei koronarer Herzkrankheit von der Gruppe M. Josephson und A. Harken aus Philadelphia [5, 6] und A.L. Waldo, Birmingham, AL [7]. Die Gruppe um J. Gallagher [8] von der Duke University in Durham, NC und RAJ Spurrell in London behandelten auch andere nichtischämische Grunderkrankungen.

**Figure 1:**
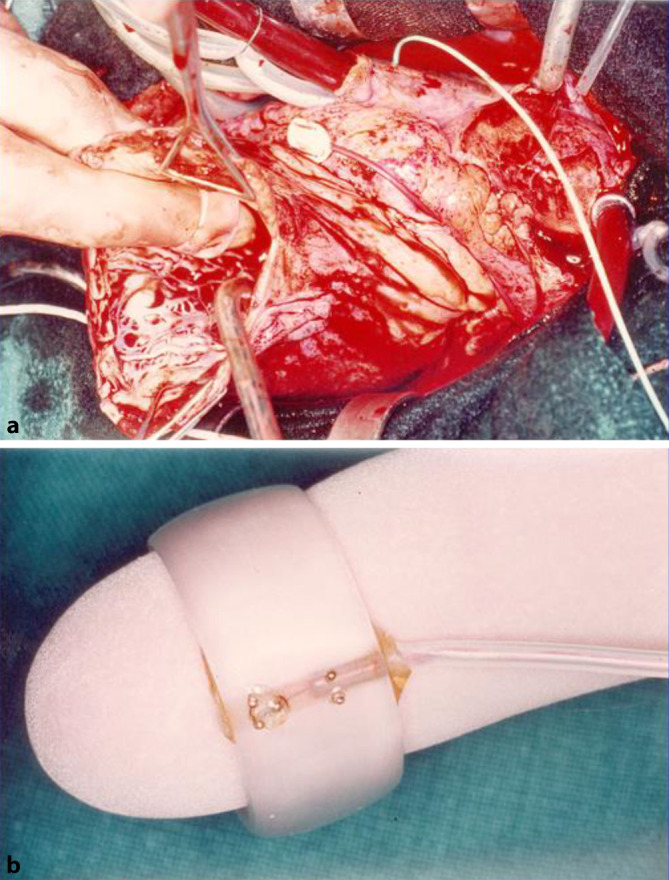

**Figure 2:**
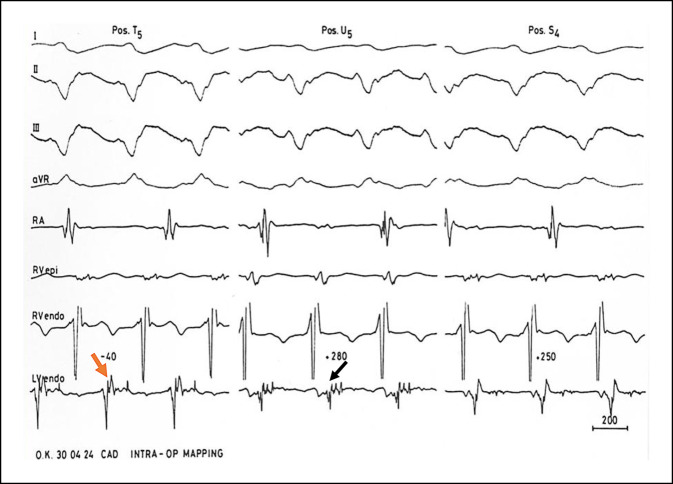

Die verschiedenen Gruppen entwickelten unterschiedliche epikardiale Mapping-Schemata und nutzten unterschiedliche Elektrodenkonfigurationen unipolarer und bipolarer Elektrogramme, aufgezeichnet mit unterschiedlichen Band-Pass-Filtern, meist zwischen 12 und 500 Hz. Die Elektroden waren zunächst meist bi- oder multipolare Stabelektroden, später wurden dann Finger-Tip-Elektroden verwendet (Abb. 1). Da es sich bei den meisten Patienten um eine koronare Grunderkrankung handelte, ging man davon aus, dass es sich bei den spontanen und induzierbaren ventrikulären Tachykardien (VT) um einen Reentry-Mechanismus der Tachykardie handeln würde, wobei die starke Verzögerung der Erregungsausbreitung in der Region der strukturellen Veränderung des Endomyokard im linken Ventrikel vermutet wurde. Dies bedeutete, dass man während Sinusrhythmus nach Elektrogrammen suchte, die durch Fraktionierung, Doppelpotentiale oder stark verzögerte Potenziale gekennzeichnet sind (Abb. 2). Bei ausgelöster stabiler VT musste dann die früheste Erregung der VT nahe an einem Mapping-Punkt mit starker Verzögerung oder Fraktionierung bei Sinusrhythmus liegen.

**Figure 3:**
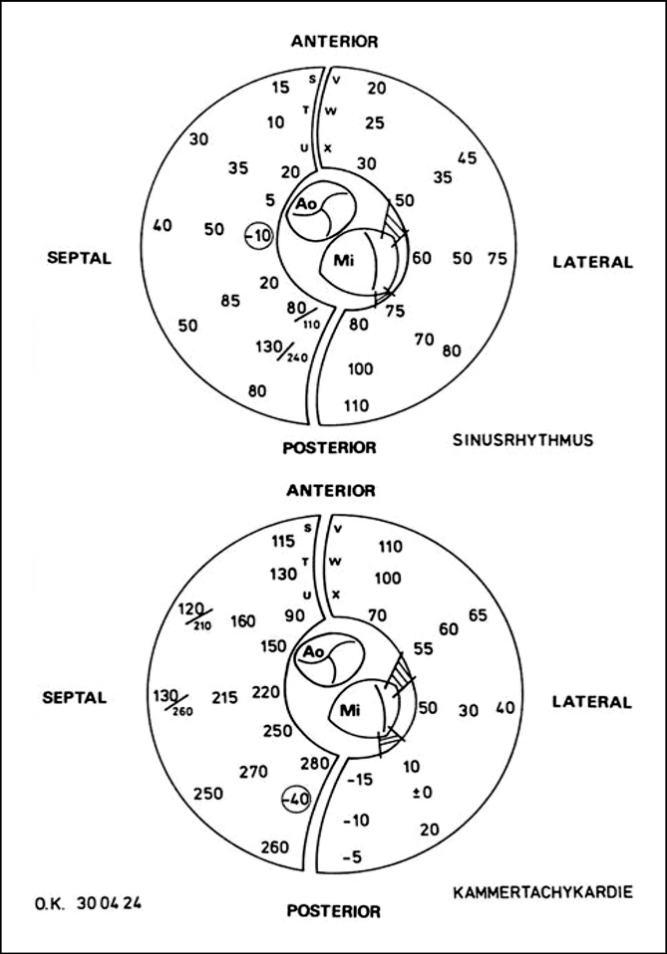

Dies war dann der Ort, der durch chirurgische Maßnahmen ausgeschaltet oder isoliert werden musste. Bei VT mit einem ektopen Tachykardie-Mechanismus war daher ein Mapping im Sinusrhythmus wenig hilfreich, und man war auf die deutlich vor dem Beginn des QRS-Komplexes der induzierten VT liegende früheste Erregung angewiesen. Als dann die präoperativen Katheter-Mapping-Verfahren Routine wurden, war die intraoperative Lokalisation des VT-Ursprungs zuverlässiger und die operative Ausschaltung des Ursprungsortes oder der kritischen Zone der VT besser möglich. Das endokardiale Mapping wurde nach Ventrikulotomie bei Normothermie während kardiopulmonalem Bypass durchgeführt. Es zeigte sich, dass der identifizierte VT-Ursprungsort entweder direkt in der Region einer erkennbaren strukturellen Veränderung des Endomyokardgewebes oder in der Randzone, d. h. am Übergang zur unveränderten Gewebestruktur lag. Es war sehr wichtig, dass beim intraoperativen „Punkt-für-Punkt-Mapping“ eine gute Zusammenarbeit zwischen dem Herzchirurgen und dem nahe dem OP-Tisch stehenden Elektrophysiologen bestand, um ein brauchbares *Mappingbild* zu erstellen. Wir mussten lernen, dass nicht selten eine Diskrepanz zwischen epikardialer und endokardialer Lokalisation eines VT-Ursprungsorts bestand (Abb. 3). Die Zeit des computergestützten Mapping mit unterschiedlicher Farbgebung der Erregungszeiten oder ein *Voltage-Mapping* zur besseren Erkennung der Gewebseigenschaft war noch nicht gekommen, d. h. ein *Carto-Computer-Mapping* oder andere später von der Industrie entwickelten Computersysteme standen nicht zur Verfügung. Interessant ist, dass Ray Ideker, damals noch an der Duke Universität in NC, USA, bereits 1979 eine computerbasierte Methode zur Darstellung der kardialen Erregungsausbreitung während des intraoperativen Mappings mit J. Gallagher entwickelt hatte [9]. Das Ziel aller chirurgischen Verfahren war immer, das *arrhythmogene* Gewebe zu entfernen, auszuschalten oder elektrisch zu isolieren.

### Chirurgische Verfahren bei der Therapie ventrikulärer Tachykardien

Man muss grundsätzlich zwei Verfahren einer chirurgischen Intervention unterscheiden, die indirekten und die direkten Verfahren. Zu den indirekten Verfahren gehören die Aneurysmektomie mit oder ohne aortokoronare Bypassoperation.

Die *indirekten Verfahren* sollen die Entstehung und Aufrechterhaltung der Arrhythmie in dem Gebiet, das für die Arrhythmie verantwortlich gemacht wird, erschweren oder verhindern. Eine solche Region ist insbesondere die Randzone zwischen unverändertem und narbig verändertem Myokard. Präoperatives oder intraoperatives Mapping gab es noch nicht. Ein erster Bericht dazu erschien von W. Sealy, der über 68 Patienten mit Aneurysmektomie wegen ventrikulärer Tachykardien berichtete. Die postoperative Mortalität betrug 35 %; bei den Überlebenden wurden 60 % als erfolgreich operiert gewertet, d. h. ohne erneute VT-Episoden. Andere Zentren berichteten über eine ähnlich hohe operative Mortalität, jedoch eine geringere Arrhythmie-Freiheit. Die zusätzliche aortokoronare Bypassprozedur bei den Aneurysmektomien änderte das Gesamtergebnis nicht. Die Gruppe an der MHH in Hannover berichtete 1979 über das Verhalten ventrikulärer Tachykardien nach einer Aneurysmektomie bei 140 Patienten, wobei 14 Patienten primär wegen medikamentös nicht beherrschbarer VT operiert wurden [10, 11]. Vier Patienten verstarben früh-postoperativ, von den 10 Überlebenden hatten zwei VT-Rezidive. Man kann daraus schließen, dass die alleinige Aneurysmektomie – mit oder ohne aortokoronarem Bypass – ohne eine gezielte Ausschaltung der Tachykardiezone kein geeignetes Verfahren zur Behandlung von rezidivierenden Kammertachykardien war.

### Direkte chirurgische Verfahren

Selbst wenn auch diese Therapieverfahren längst Geschichte sind, und sie von den Katheterablationsverfahren abgelöst wurden, erscheint es sinnvoll, diese Verfahren und ihre Ergebnisse einmal zu beleuchten. Dabei soll die Geschichte der Interventionen zur Durchtrennung von akzessorischen Leitungsbahnen abgetrennt werden von den unterschiedlichen Techniken zur Behandlung von ventrikulären Tachykardien.

#### Encircling Endocardial Ventriculotomy (EEV)

Dieses Verfahren, das erstmals von G. Guiraudon et al. 1978 beschrieben wurde [4], versuchte, *kritisches*, für die VT-Entstehung und Aufrechterhaltung verantwortlich gemachtes Gewebe zirkulär durch eine vom Endokard ausgeführte senkrechte Inzision, bis fast zum Epikard hindurchreichend, vom unveränderten Myokardgewebe abzutrennen. In der ersten Beschreibung von G. Guiraudon waren 18 von 22 Patienten nach der EEV frei von ventrikulären Tachykardien; zwei Patienten überlebten den Eingriff nicht. Intraoperatives Mapping wurde nicht durchgeführt; postoperative Angaben zur Ventrikelfunktion fehlen. Später wurde die vollständige zirkuläre Ventrikulotomie in eine *semizirkuläre* Inzision abgewandelt, was besonders bei einer septalen Narbe, oder bei Involvierung der Papillarmuskelansätze vorteilhafter erschien [11].

#### Endokardiale Resektion (Endokardial Peel-off)

Fast gleichzeitig mit der Gruppe um G. Guiraudon haben M. Josephson und A. Harken in Philadelphia über eine Methode der endokardialen Resektion („Peel-off“ 1–2 mm tief) des Arrhythmiegewebes, das durch intraoperatives Mapping bei Sinusrhythmus und induzierter VT identifiziert worden war, berichtet [5, 6]. Zwei von 29 Patienten starben innerhalb von 24 h postoperativ, drei verstarben später. Bei 24 von 27 Patienten konnte nach dem Eingriff keine VT ausgelöst werden. Die Arbeitsgruppe um A.L. Waldo und J. Kirklin in Birmingham, AL, USA benutzte bei den ersten 38 Patienten nur das intraoperative Mapping-Ergebnis während Sinusrhythmus mit ausgeprägter Fraktionierung der Elektrogramme, Doppelpotentialen oder „delayed potentials“ zur Identifikation der Arrhythmieregion [12]. Die Resultate waren vergleichbar mit denen der Philadelphia-Gruppe um M. Josephson. Eine Gruppe um P.V.L. Curry in London benutzte die 12-Kanal-EKG-Ableitung der prä-operativ induzierten VT, um diese mit einem intraoperativ durchgeführten *Pace-Mapping* zu vergleichen und führte dann in dieser Region eine Endokardresektion durch. J. Gallagher und W. Sealy von der Duke Universität in Durham, NC, USA, sowie A.J. Camm und R.A.J. Spurrell in London [13] waren 1979 und 1980 die ersten Zentren, die kryochirurgische Technologie intraoperativ anwendeten, um das identifizierte Arrhythmie-Areal auszuschalten. 1986 berichteten J.G. Selle und W. Sealy aus Charlotte, NC, USA erstmals über eine erfolgreiche Anwendung von YAG-Laser-Technologie zum intraoperativen Ausschalten des „Arrhythmiegewebes“ [14]. G. Breithardt (Münster) hat in einer persönlichen Mitteilung berichtet, dass 1988 einige VT-Patienten in Münster mit der Laser-Technologie behandelt wurden. Dabei hätte er die von M. Borggrefe identifizierten Arrhythmie-Areale mit Hilfe eines Laserpointers unsteril vom Kopfende des OP-Tisches her dem Chirurgen gezeigt, wo die Laserablation durchzuführen sei.

Die Ergebnisse der operativen Behandlung von Kammertachykardien in den ersten 3 Jahren (1978–1981) sind nur schwer vergleichbar, weil jeweils nur über kleine Fallzahlen bei einem heterogenen Patientenkollektiv und unterschiedlichen Grunderkrankungen berichtet wurde – z. T. auch nur in Abstract-Form. Dies erklärt auch, warum sich letztlich nur wenige Zentren weiter mit der chirurgischen Ausschaltung von Kammertachykardien beschäftigten, zumal auch herzchirurgische Zentren mit speziellem Interesse für diese Operationsverfahren bereitstehen mussten.

### Chirurgische Behandlung von Kammertachykardien in Deutschland

Wenn man an Hand von Publikationen versucht zu ermitteln, wie verbreitet die chirurgischen Therapieverfahren bei Tachyarrhythmien in Deutschland seit 1980 waren, scheint es, dass nur die Universitätskliniken Düsseldorf und später Münster und die Universitätskliniken der Medizinischen Hochschule Hannover beteiligt waren. Das schließt nicht aus, dass auch andere Kliniken in Deutschland, der Schweiz oder Österreich chirurgische Interventionen bei Tachykardien durchgeführt haben. Für die Medizinische Hochschule Hannover können die Autoren bestätigen, dass andere Kliniken wie die Universitätsklinik Bonn (Prof. M. Manz) und sogar einzelne Kliniken in Italien Patienten zur Behandlung an die Medizinische Hochschule Hannover überwiesen haben.

Die Zeitspanne der chirurgischen Therapien von Tachyarrhythmien in Deutschland war kurz; sie wurde von zunehmender Anwendung der Defibrillatortherapie einerseits (seit 1984) und der aufkommenden Katheterablation andererseits eingegrenzt.

**Figure 4:**
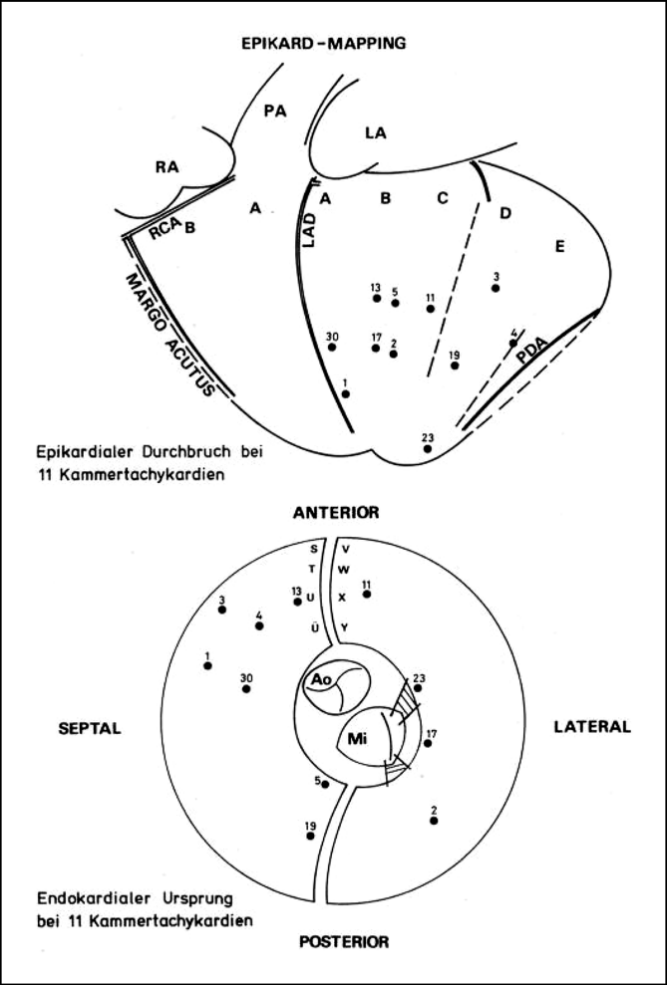

Die Düsseldorfer Gruppe um G. Breithardt, L. Seipel, M. Borggrefe und dem Herzchirurgen J. Ostermeyer begann 1978 mit der chirurgischen Therapie von ventrikulären Tachykardien [15, 16]. Über ihre operative Technik und erste Ergebnisse wurde 1979 berichtet. Zwei Jahre später hat dann die Hannover-Gruppe mit G. Frank und H. Klein ihre ersten Ergebnisse zur VT-Chirurgie veröffentlicht [17], nachdem H. Klein von seinem 2‑jährigen Forschungsaufenthalt bei A.L. Waldo in Birmingham, AL, USA zurückgekehrt war und G. Frank sich ebenfalls für einige Monate bei dem bekannten Herzchirurgen J. Kirklin und N. Kouchoukos am UAB in Birmingham die chirurgische Technik der VT-Chirurgie angeeignet hatte. Es wurde über 19 Patienten berichtet, bei denen nach prä- und intraoperativem epikardialem und endkardialem Mapping eine endokardiale zirkuläre Inzision nach der Methode Guiraudon durchgeführt wurde. Postoperativ konnte keine VT ausgelöst werden; ein Patient hatte innerhalb eines Jahres nach dem Eingriff ein VT-Rezidiv, 4 (21 %) verstarben in diesem Zeitraum durch kardiales Pumpversagen. 1982 und 1984 veröffentlichte die Düsseldorfer Gruppe mit J. Ostermeyer, G. Breithardt und M. Borggrefe ihre Ergebnisse über 40 behandelte Patienten, die zwischen 1978 und 1983 operiert wurden [18–20]. Eine wichtige Information dieser Publikationen war, dass eine nur *semizirkuläre* endokardiale Inzision ebenso effektiv war (70 %), wie eine komplette zirkuläre Inzision; aber die Beeinträchtigung der LV-Pumpfunktion war deutlich geringer (8 % versus 46 %), und auch die Langzeitmortalität war mit 8 % gegenüber 11 % geringer. Später, 1987, berichtete J. Ostermeyer aus der Düsseldorfer Gruppe über die Ergebnisse von 93 operierten Patienten [21]; dabei war bei 19 % früh postoperativ eine VT induzierbar, nach einem Jahr blieben 87 %, nach 5 Jahren 77 % ohne VT-Rezidive; früh postoperativ verstarben 5 %, die Mortalität nach einem Jahr war 11 und 30 % nach 5 Jahren. Die Autoren schlussfolgerten aus ihren Ergebnissen, dass eine partielle endokardiale Inzision vorteilhafter sei als die endokardiale Resektion. Die Hannover-Gruppe mit H‑J Trappe, G. Frank und H. Klein führte bis 1992 eine chirurgische Therapie bei rezidivierenden Kammertachykardien bei 147 Patienten durch [22–24]; 93 % hatten eine koronare Herzkrankheit mit Zustand nach Myokardinfarkt, 11 (7 %) hatten eine rechts-/linksventrikuläre Dysplasie. Bei der Mehrzahl der Operierten wurde nach 1984 sowohl ein präoperatives Katheter-Mapping zur Lokalisation des VT-Ursprungsorts als auch ein intraoperatives epikardiales und endokardiales Mapping im Sinusrhythmus und bei induzierter VT bei normothermer (37 Grad) Perfusionstemperatur durchgeführt (Abb. 4). Bei etwa der Hälfte der operierten Patienten nach Myokardinfarkt lagen Teile des LV-Septums im identifizierten Tachykardie-Areal. Bei den Patienten mit RV/LV-Dysplasie lag der Ursprung der Tachykardie in 70 % der Fälle im rechten Ventrikel, bei 30 % war zusätzlich der linke Ventrikel oder das LV-Septum der Ursprungsort der VT. Im Gegensatz zur Düsseldorfer Gruppe wurde in Hannover nach den ersten, eher mäßigen Ergebnissen mit der endokardialen Inzision dann nur noch die subendokardiale Resektion („Peel-off“) nach Josephson und Harken durchgeführt; bei inferiorem oder septalem VT-Ursprung, oder Beteiligung der Papillarmuskeln, wurde zusätzlich oder ausschließlich eine Multi-Punkt-Kryoablation bei minus 80–90 Grad angewandt. Bei den RV-Dysplasien wurde nur eine ausgedehnte Kryoablation durchgeführt. Bei 9 % aller Patienten mit subendokardialer Resektion war intraoperativ wieder eine VT induzierbar, sodass nach erneutem Mapping eine weitere Endokardresektion durchgeführt wurde. Bei 31 % der Koronarpatienten erfolgte zusätzlich eine Bypassoperation und bei 48 % eine Aneurysmaresektion. Die frühe postoperative Mortalität durch kardiales Pumpversagen lag bei 7 %. Nach einer mittleren Nachbeobachtung von 37 Monaten waren 37 Patienten (27 %) verstorben, 9 (6 %) verstarben plötzlich, 23 (17 %) durch kardiales Pumpversagen und 4 % durch nichtkardiale Ursachen. Bei 24 Patienten (18 %) kam es nach Krankenhausentlassung zu VT-Rezidiven; bei 18 von 125 (14 %) mit koronarer Herzerkrankung und bei 6 von 11 Patienten mit ARVD (55 %). Bei 21 von 24 Patienten (87 %) wurde danach ein ICD implantiert.

Eine wichtige Übersicht zur Methodik, Patientenselektion, Rezidivrate, Komplikationsrate und Mortalität der chirurgischen Therapie von Kammertachykardien publizierte M. Borggrefe mit Daten eines internationalen Registers zur antitachykarden VT-Chirurgie bei 665 Patienten aus 8 Zentren weltweit (Düsseldorf, Hannover, Philadelphia mit M. Josephson, Newark mit S. Saksena, Stanford mit C. Swerdlow, Paris mit G. Fontaine, Newcastle upon Tyne mit R.W. Campbell und Pavia mit J. Salerno; [25]). Das Register lieferte wichtige Informationen zu prä- und intraoperativen Mappingverfahren; bei der Mehrheit wurde eine begrenzte Endokardresektion (ER) durchgeführt (33 %), gefolgt von der semizirkulären Inzision, wie sie von Düsseldorf eingeführt wurde, einer Kombination von begrenzter ER und kryochirurgischen Verfahren (16 %), und bei 13 % wurde die Laser-Technologie verwendet. Bei den nichtkoronaren Kardiomyopathien wurde fast immer die Kryochirurgie-Technik verwendet. Bei den Koronarpatienten erfolgte in 50 % aller Patienten eine zusätzliche ACVB-Operation und bei 70 % eine Aneurysmektomie. Die geringste perioperative Mortalität wurde mit der semizirkulären Endokardinzision erreicht (8 %), gefolgt von der Kryochirurgie (8,5 %) und der begrenzten Endokardresektion (10 %). Eine ausgedehnte Endokardresektion hatte mit 27 % die höchste perioperative Mortalität. Die Langzeitüberlebensrate nach 2 Jahren war 72 % und nach 5 Jahren bei 57 %, 18 Patienten (2,8 %) verstarben plötzlich. Patienten ohne Koronarerkrankung hatten eine deutlich geringere Langzeitmortalität. Die früh-postoperative VT-Rezidivrate der den Eingriff überlebenden Patienten lag bei 8 %, während bei der Langzeit-Nachbeobachtung (im Mittel 27 Monate) bei 68 Patienten (10,5 %) erneut Kammertachykardien auftraten.

Zusammenfassend muss man feststellen, dass die relativ kurze Geschichte der chirurgischen Therapieverfahren bei Kammertachykardien ein Versuch war, das Problem der wenig wirksamen Antiarrhythmika zu überwinden. Die prä- und intraoperativen Mappingverfahren haben das Verständnis des Arrhythmie-Mechanismus verbessert, die programmierte Ventrikelstimulation (PVS) erwies sich als hilfreich zur Voraussage einer chirurgischen Interventionsmöglichkeit und erlaubte relativ zuverlässig die längerfristige Vorhersage einer erfolgreichen Intervention oder des Risikos eines VT-Rezidivs. Eine relativ hohe operative und Langzeit-Mortalität wurde zunächst als akzeptabel angesehen.

Fast gleichzeitig mit der Verbesserung der chirurgischen Therapieverfahren entwickelte sich einerseits die ICD-Therapie mit den Möglichkeiten der antitachykarden Stimulation und transvenösen Implantation der Elektroden; andererseits hatte sich die aus einem *elektrischen Unfall* bei G. Fontaine in Paris geborene Katheterablation, die zunächst mit DC-Schocks durchgeführt wurde und die sehr schnell durch die Radiofrequenz(HF)-Katheterablation abgelöst wurde, einen festen Platz bei der Behandlung von Tachykardien gesichert und damit die aufwendigen chirurgischen Verfahren verdrängt. Dazu beigetragen hat die enorm hilfreiche Verbesserung des Mappings mit der computergestützten Bildgebung bei der Lokalisation des arrhythmogenen Areals.

So bleibt die chirurgische Therapie der Herzrhythmusstörungen, die letztlich weltweit auch nur von wenigen Zentren angewandt wurde, eine kurze Episode in der Geschichte der Rhythmologie.

## *Teil 2:* Chirurgische Behandlung supraventrikulärer Tachykardien, insbesondere Präexzitationssyndromen (WPW)

Der Beginn der chirurgischen Durchtrennung einer atrioventrikulären akzessorischen Leitungsbahn begann 1968, also früher als die ersten Versuche einer chirurgischen Ausschaltung von Kammertachykardien. Durch die Verbesserung der präoperativen elektrophysiologischen Diagnostik mit mehreren Elektrodenkathetern im rechten Vorhof, der His-Bündel-Region, die Positionierung von Multi-Elektroden-Kathetern im Koronarsinus und im rechten Ventrikel konnte eine akzessorische Leitungsbahn recht genau lokalisiert werden und nach Induktion der supraventrikulären Tachykardie die antegrade und retrograde Leitungseigenschaft lokalisiert und analysiert werden.

Grundsätzlich wurden zwei unterschiedliche operative Techniken und Zugangswege zur Unterbrechung der genau lokalisierten Leitungsbahnen angewendet. Eine endokardiale Vorgehensweise wurde von W. Sealy (Duke University in Durham, NC, USA) bereits 1968 beschrieben und von der Gruppe um J. Gallagher und J. Cox bis 1990 weitergeführt [26, 27]. Die epikardiale Vorgehensweise wurde von G. Guiraudon in Paris – später in London, Ontario, Kanada − beschrieben und weiterverfolgt [28]. Beide Verfahren benötigten zunächst eine mediane Sternotomie. Die endokardiale Vorgehensweise erforderte den Gebrauch der extrakorporalen Zirkulation bei normaler Körpertemperatur während des epikardialen und endokardialen Mappings, und für die Inzision des Endokards eine Temperatursenkung auf etwa 34 Grad im kardioplegischen Stillstand. Dagegen benötigte der epikardiale Zugang keinen kardioplegischen Stillstand und wurde am schlagenden Herzen durchgeführt. Mehrheitlich haben später die meisten Zentren die epikardiale Vorgehensweise genutzt und die Unterbrechung des akzessorischen Bündels mit einer kryochirurgischen Maßnahme kombiniert.

Für die anterior-septalen und posterior-septalen Leitungsbahnen konnte dagegen nur der endokardiale Zugang gewählt werden. Die endokardiale Prozedur war eine Kombination von atrialer Inzision entlang des atrioventrikulären Anulus in der Region der ermittelten Leitungsbahn.

Allerdings berichteten dann J. Bredikis et al. 1985 über eine Methode zur Durchtrennung rechtslateraler und septaler Leitungsbahnen ohne extrakorporale Zirkulation durch Anwendung von Kryotechnologie [29]. Für die linkslateralen und linksposterioren Leitungsbahnen erfolgte die atriale Inzision semizirkulär entlang des Mitralklappensulkus. Bei den posterior-septalen, den rechtslateralen und den anterior-septalen Bündeln erfolgte nach rechtsatrialer Inzision eine Dissektion entlang des Trikuspidalklappen-AV-Sulkus.

Die epikardiale Vorgehensweise bestand aus einer kompletten epikardialen Dissektion des Koronarsulkus vom epikardialen Fettgewebe mit sorgfältiger Isolation der Koronararterien. Bei etwa 7–10 % der WPW-Syndrome gibt es multiple akzessorische Leitungsbahnen, häufig posterior-septal. Das erforderte manchmal sowohl den epikardialen als auch den endokardialen Zugangsweg. Nicht selten waren akzessorische Leitungsbahnen mit kongenitalen Veränderungen verbunden; besonders häufig mit einer Ebstein-Anomalie.

G. Guiraudon berichtete über 502 Patienten, die bis zum Beginn der Katheterablation von Tachykardien 1990 operiert wurden. Von 1990–1993 waren es dann noch einmal 370 Patienten, bei denen die Katheterablation erfolglos blieb oder zusätzliche chirurgische Verfahren erforderlich waren [30].

In Deutschland sind nach Kenntnis der Autoren chirurgische Therapieverfahren beim Präexzitationssyndrom nur in 2 Zentren – von 1980 bis zum Einsatz der RF-Katheterablation ab 1990 durchgeführt bzw. publiziert worden. Das schließt nicht aus, dass einzelne Fälle auch in anderen Zentren mit Herzchirurgie und Erfahrung mit dem WPW-Syndrom operiert wurden, ohne dass Publikationen dazu vorliegen.

Die Düsseldorfer Gruppe um L. Seipel, J. Ostermeyer, G. Breithardt und M. Borggrefe haben erstmals 1979/1980 über intraoperative Mappingverfahren bei chirurgischer Therapie des WPW-Syndroms berichtet [31]. Interessant ist der erste Bericht von L. Seipel 1980 über eine intraoperative Durchtrennung eines nur retrograd leitenden akzessorischen Bündels während einer Aortenklappenoperation [32].

M. Borggrefe berichtete dann über die Düsseldorfer Erfahrung mit der chirurgischen Therapie des WPW-Syndroms mit Vorhofflimmern bei 18 Patienten [33]. Die Mehrheit der Patienten hatte ein linkslaterales Bündel, das nach der W.-Sealy-Methode durchtrennt wurde. Bei 5 Patienten wurde zusätzlich eine Klappenoperation durchgeführt; ein Patient mit einer Ebstein-Anomalie verstarb bei diesem Eingriff, 3 Operierte benötigten einen zweiten Eingriff. Nach im Mittel 26 Monaten hatten 14 der 18 Operierten keine weiteren Tachykardien, bei den Übrigen waren die Tachykardien dann gut mit Medikamenten unterdrückbar. Düsseldorf/Münster hat sich dann nach 1987 sehr schnell auf die HF-Katheterablation des WPW-Syndroms konzentriert.

Eine erste Patientin aus Hannover wurde 1982 gemeinsam mit W. Sealy und J.J. Gallagher in Charlotte, NC, USA operiert. Es war eine junge Turnierreiterin, die nach einem Herzstillstand bei tachykardem Vorhofflimmern reanimiert werden konnte. G. Frank und H. Klein sind dann mit der Patientin nach Charlotte geflogen, um die operative Technik der WPW-Behandlung zu erlernen. Obwohl die junge Frau innerhalb von 24 h dreimal operiert wurde, um die akzessorische Leitung erfolgreich zu durchtrennen, erschreckte es G. Frank nicht, sich in Hannover auf die chirurgische Therapie von Tachykardien zu konzentrieren.

Im Jahr 1989 hat die Hannover-Gruppe mit G. Frank, H. Klein und dem Kinderkardiologen C. Kallfelz über die chirurgische Therapie von nicht mit Medikamenten beherrschbaren Tachykardiesyndromen bei 10 Kindern im Alter zwischen 2 und 14 Jahren berichtet [34]. Bei 7 Kindern bestand ein WPW-Syndrom, bei 2 Kindern rechts- bzw. linksatriale fokale Tachykardien und bei einem Kind mit Fallot-Tetralogie eine ventrikuläre Tachykardie. In allen Fällen wurde die Kryoablationstechnik angewandt. Zwei Kinder benötigten nach wenigen Tagen einen zweiten Eingriff, ein Kind benötigte danach eine Schrittmacher-Implantation. Neun von 10 Kindern blieben ohne weitere Tachykardien bis ins Erwachsenenalter; bei dem Kind mit der Fallot-Tetralogie konnten danach die Tachykardien mit Antiarrhythmika beherrscht werden.

Eine interessante Erfahrung machte die Hannover-Gruppe, als sie 1987 noch zur DDR-Zeit ganz offiziell nach Leipzig eingeladen wurde, um dort einige Patienten mit Tachykardien zu operieren. Dies war ein ganz besonderes Erlebnis, denn so konnte bereits 2 Jahre vor dem Mauerfall eine ganz persönliche Freundschaft mit einigen Rhythmologen der DDR aufgebaut werden.

Im Jahr 1993 hat die Hannover-Gruppe ihre Erfahrungen und Langzeitergebnisse der chirurgischen Therapie von supraventrikulären Tachykardien zwischen 1984 und 1992 bei 120 Patienten vorgestellt, die wegen eines WPW-Syndroms mit medikamentös nicht unterdrückbaren Tachykardien operiert wurden [35]. Es waren 78 Männer und 42 Frauen, mittleres Alter 36 Jahre. Bei 63 % der Operierten lagen die akzessorischen Bündel linkslateral und linksposterior, bei 24 % rechtslateral und rechtsanterior und bei 13 % im membranösen Teil des interventrikulären Septums. Bei den linkslateralen akzessorischen Bündeln erfolgte der Zugang zum Herzen in vielen Fällen über eine posterior-laterale Thorakotomie mit epikardialer Dissektion im AV-Sulkus – ohne Gebrauch einer extrakorporalen Zirkulation. Bei 95 % aller lokalisierten akzessorischen Bahnen wurde zusätzlich eine Kryoablation durchgeführt. Bei 105 von 120 Patienten (88 %) war die operative Behandlung erfolgreich, d. h. ohne erneute Tachykardien mit einer mittleren Nachbeobachtungszeit von 42 Monaten. Bei 12 Patienten erfolgte nach Wiederauftreten einer Delta-Welle oder Tachykardien eine Reoperation. Zwei Patienten verstarben früh-postoperativ nach der Reoperation durch eine Sepsis und ein Rechtsherzversagen bei einer Ebstein-Anomalie. Zehn der 12 erneut operierten Patienten blieben dann ohne Tachykardie-Rezidiv und ohne Antiarrhythmika.

Die Geschichte der chirurgischen Therapie bei supraventrikulären Tachykardien ist noch kürzer als die der chirurgischen Verfahren bei ventrikulären Tachykardien. Die Entwicklung der Katheterablation hat beide chirurgischen Verfahren abgelöst, insbesondere als die DC-Katheterablation nach kurzer Zeit durch die RF-Ablation ersetzt wurde, und gleichzeitig die digitalen Mappingsysteme während des Katheter-Mappings die Lokalisation der akzessorischen Leitungsbahnen stark verbessert hatten. Die letzte Phase einer Entwicklung zur chirurgischen Behandlung von Vorhofflimmern ist die sogenannte MAZE-Prozedur, die 1991 von J. Cox et al. (St Louis, MO, USA) beschrieben wurde und die von 1987 bis 1991 an 7 Patienten durchgeführt wurde [36]. Dabei wird der linke Vorhof bei einem herzchirurgischen Eingriff durch eine *Labyrinth-artige* Schnittführung so verändert, dass die aus den Pulmonalvenen in den linken Vorhof gelangenden Impulse kein dauerhaftes Vorhofflimmern auslösen können. Eine große Verbreitung hat dieses chirurgische Verfahren nicht erlangt.

So bleibt auch die Chirurgie der supraventrikulären Tachykardien ein kurzer Abschnitt in der Geschichte der Rhythmologie. Diese Verfahren wurden nur von wenigen Zentren durchgeführt.

## References

[1] Couch OA (1959). Cardiac aneurysm with ventricular tachycardia and subsequent excision of aneurysm. (Case report). Circulation.

[2] Fontaine G, Guiraudon G, Frank R, Gerhaux A, Cousteau Barillon JRA (1975). La cartographie épicardique et le traitement chirurgical par simple ventriculotomie de certaines tachycardies ventriculaires rebelles par reentrée. Arch Mal Coeur Vaiss.

[3] Fontaine G, Guiraudon G, Frank R, Coutt R, Dragodanne C, Wellens HJJ, Lie KI, Janse MJ (1976). Epicardial mapping and surgical treatment in six cases of resistant ventricular tachycardia not related to coronary artery disease. The Conduction System of the Heart. Structure, Function, and Clinical Implications.

[4] Sealy WC (1979). Direct surgical treatment of arrhythmias: The last frontier in surgical cardiology. Chest.

[5] Josephson ME, Harken AH, Horowitz LN (1979). Endocardial excision—a new surgical technique for the treatment of recurrent ventricular tachycardia. Circulation.

[6] Harken AH, Horowitz LN, Josephson ME (1979). Endocardial excision guided by ventricular mapping in the surgical treatment of ventricular tachycardia. Am J Cardiol.

[7] Waldo AL, Arciniegas JG, Klein H (1981). Surgical treatment of life-threatening ventricular arrhythmias. The role of intraoperative mapping and consideration of the presently available surgical techniques. Prog Cardiovasc Dis.

[8] Gallagher JJ (1978). Surgical treatment of arrhythmias: current status and future directions. Am J Cardiol.

[9] Ideker RE, Smith WM, Wallace AG, Kasell J, Harrison LA, Klein GJ, Kinicki RE, Gallagher JJ (1979). A computerized method for the rapid display of ventricular activation during the intraoperative study of arrhythmias. Circulation.

[10] Klein H, Bethge K-P, Frank G, Borst H-G, Lichtlen P (1979). Das Verhalten ventrikulärer Arrhythmien nach Aneurysmektomien. Z Kardiol.

[11] Frank G, Spieker K, Klein H (1983). Lebenserwartung und Spätergebnisse nach Resektion von post-infarziellen Herzwandaneurysmen. Leb Heft.

[12] Klein H, Karp RB, Kouchoukos NT, Zorn GL, James TN, Waldo AL (1982). Intraoperative electrophysiologic mapping of the ventricles during sinus rhythm in patients with a previous myocardial infarction. Identification of the electrophysiologic substrate of ventricular arrhythmia. Circulation.

[13] Camm J, Ward DE, Spurrell RAJ, Rees GM (1980). Cryothermal mapping and cryoablation in the treatment of refractory cardiac arrhythmias. Circulation.

[14] Selle JG, Svenson RH, Sealy WC (1986). Successful clinical laser ablation of ventricular tachycardia: a promising new therapeutic method. Ann Thorac Surg.

[15] Ostermeyer J, Breithardt G, Kolvenbach R, Abendroth RR, Seipel L, Bircks W (1979). Electrophysiological mapping during open-heart surgery. Z Kardiol.

[16] Abendroth RR, Ostermeyer J, Breithardt G, Seipel L, Bircks W (1980). Reproducibility of local activation times during intraoperative epicardial mapping. Circulation.

[17] Frank G, Klein H, Lichtlen P, Borst HG (1981). Direct surgical therapy of ventricular arrhythmias in coronary heart disease. Thorac Cardiovasc Surg.

[18] Ostermeyer J, Breithardt G, Kolvenbach R, Borggrefe M, Seipel L, Bircks W (1982). The surgical treatment of ventricular tachycardias: simple aneurysmectomy versus electrophysiologically guided procedures. J Thorac Cardiovasc Surg.

[19] Ostermeyer J, Breithardt G, Borggrefe M, Godehardt E, Seipel L, Bircks W (1984). Surgical treatment of ventricular tachycardias Complete versus partial encircling endocardial ventriculotomy. J Thorac Cardiovasc Surg.

[20] Breithardt G, Borggrefe M, Ostermeyer J, Seipel L, Bircks W (1984). Clinical and electrophysiologic findings following operative therapy of ventricular tachycardias. Z Kardiol.

[21] Ostermeyer J, Borggrefe M, Breithardt G, Podczek A, Goldmann A, Schoenen JD, Kolvenbach R, Godehardt E, Kirklin JW, Blackstone EH (1987). Direct operations for the management of life-threatening ischemic ventricular tachycardia. J Thorac Cardiovasc Surg.

[22] Trappe HJ, Klein H, Frank G, Wenzlaff P, Lichtlen PR (1992). Role of mapping-guided surgery in patients with recurrent ventricular tachycardia. Am Heart J.

[23] Trappe HJ, Klein H, Wenzlaff P, Frank G, Siclari F, Fieguth H-G, Wahlers Th, Lichtlen PR (1993). Langzeitverlauf nach antitachykarder Operation bei Patienten mit Kammertachykardien. Med Klin.

[24] Trappe HJ, Pfitzner P, Fieguth HG, Wenzlaff P, Kielblock B, Klein H (1994). Nonpharmacological therapy of ventricular tachyarrhythmias: observations in 554 patients. Pacing Clinical Electrophis.

[25] Borggrefe M, Podczeck A, Ostermeyer J, Breithardt G, Breithardt G, Borggrefe M, Zipes DPZ (1987). Long-term results of electrophysiologically guided antitachycardia surgery in ventricular tachyarrhythmias: A collaborative report on 665 patients. Nonpharmacological therapy of tachyarrhythmias.

[26] Sealy WC, Hattler BG, Blumenschein SD, Cobb FR (1969). Surgical treatment of Wolff-Parkinson-White Syndrome. Ann Thorac Surg.

[27] Sealy WC, Gallagher JJ, Wallace AG (1976). The surgical treatment of Wolff-Parkinson-White syndrome: evolution of improved methods for identification and interruption of the Kent bundle. Ann Thorac Surg.

[28] Guiraudon GM, Klein GJ, Gulamhusein S, Jones DL, Yee R, Perkins G, Jarvis E (1984). Surgical repair of Wolff-Parkinson-White Syndrome: A new closed-heart technique. Ann Thorac Surg.

[29] Bredikis J, Bukauskas F, Zebrauskas R (1985). Crysurgical ablation of right parietal and septal accessory atrioventricular connections without the use of extracorporeal circulation. J Thorac Cardiovasc Surg.

[30] Guiraudon GM, Klein GJ, Yee R (1993). Supraventricular tachycardia: the role of surgery. Pacing Clin Electrophysiol.

[31] Ostermeyer J, Breithardt G, Kolvenbach R, Körfer R, Seipel L, Schulte HD, Bircks W (1979). Intraoperative electrophysiologic mapping during cardiac surgery. Thorac Cardiovasc Surg.

[32] Seipel L, Ostermeyer J, Abendroth RR, Bircks W, Breithardt G, Gleichmann U (1980). Demonstration of retrograde block in a patient with pre-excitation syndrome by intraoperative electrophysiological studies. Z Kardiol.

[33] Borggrefe M, Breithardt G, Ostermeyer J, Bircks W (1987). Surgical treatment of Wolff-Parkinson-White Syndrome. Z Kardiol.

[34] Frank G, Schmid C, Baumgart D, Lowes H, Klein H, Kallfelz HC (1989). Surgical therapy of life-threatening tachycardic cardiac arrhythmias in children. Monatsschr Kinderheilkd.

[35] Lowes D, Frank G, Klein H, Manz M (1993). Surgical treatment of the Wolff-Parkinson-White Syndrome-Experience in 120 patients. Eur Heart J.

[36] Cox JL, Schuessler RB, D’Agostino HJ, Stone CM, Chang B-C, Cain ME, Corr PB, Boineau JP (1991). The surgical treatment of atrial fibrillation. J Thorac Cardiovasc Surg.

